# Metabolic network of the gut microbiota in inflammatory bowel disease

**DOI:** 10.1186/s41232-024-00321-w

**Published:** 2024-03-05

**Authors:** Kohei Sugihara, Nobuhiko Kamada

**Affiliations:** 1 WPI Immunology Frontier Research Center, Osaka University, Suita, Osaka, Japan; 2 Division of Gastroenterology and Hepatology, Department of Internal Medicine, University of Michigan, 1150 W. Medical Center Drive, Ann Arbor, MI 48109 USA; 3 Department of Pathology, University of Michigan, Ann Arbor, MI USA

**Keywords:** Gut microbiota, IBD, Pathobiont, AIEC, Metabolic network

## Abstract

Gut dysbiosis is closely linked to the pathogenesis of inflammatory bowel disease (IBD). Emerging studies highlight the relationship between host metabolism and the modulation of gut microbiota composition through regulating the luminal microenvironment. In IBD, various disease-associated factors contribute to the significant perturbation of host metabolism. Such disturbance catalyzes the selective proliferation of specific microbial populations, particularly pathobionts such as adherent invasive *Escherichia coli* and oral-derived bacteria. Pathobionts employ various strategies to adapt better to the disease-associated luminal environments. In addition to the host-microbe interaction, recent studies demonstrate that the metabolic network between commensal symbionts and pathobionts facilitates the expansion of pathobionts in the inflamed gut. Understanding the metabolic network among the host, commensal symbionts, and pathobionts provides new insights into the pathogenesis of IBD and novel avenues for treating IBD.

## Background

Inflammatory bowel disease (IBD) is a chronic and relapsing inflammatory disease of the gastrointestinal tract that includes ulcerative colitis (UC) and Crohn’s disease (CD). Given that the emergence of IBD in developing countries over the past 25 years suggests that this epidemiologic evolution of IBD is related to the westernization of lifestyle and industrialization [1]. According to epidemiologic studies, diet, antibiotics, hygiene status, and breastfeeding have been implicated as potential environmental risk factors for IBD [2, 3]. These environmental factors influence the composition and functions of the gut microbiota, which are associated with the pathogenesis of IBD. Therefore, manipulation of gut microbiota by dietary intervention, prebiotics, probiotics, and fecal microbiota transplantation (FMT) is being developed for the treatment of IBD [4–7]. However, the efficacy of gut microbiota-targeted therapies in patients with IBD is limited. To improve therapeutic efficacy, a deeper understanding of the role of gut microbiota in IBD is needed. In this review, we discuss the complex interactions between the host and the gut microbiota in the context of IBD. In particular, we focus on the metabolic networks between the host and gut microbiota during gut inflammation and how metabolic networks influence the expansion of IBD-associated pathobionts. We also highlight the future direction of treatment targeting the gut microbiota in IBD.

### Gut microbiota and IBD

#### The role of gut microbiota in IBD

A wide variety of microorganisms inhabit the gastrointestinal tract, forming a complex microbial ecosystem. Gut microbiota has a variety of physiological functions, such as degradation of dietary-derived nutrients, production of vitamins and other nutrients, development of the intestinal immune system, and inhibition of pathogenic bacteria. On the other hand, the host provides the gut microbiota with the nutrients and space for their growth, forming a symbiotic relationship between the host and the gut microbiota. This symbiotic relationship between gut microbiota and the host plays a vital role in maintaining intestinal homeostasis [8, 9]. However, the symbiotic relationship is disrupted by gut inflammation, leading to gut dysbiosis characterized by the blooms of pathobionts and the reduction of beneficial commensals.

Accumulating evidence suggests that gut microbiota plays a vital role in the pathogenesis of IBD [10, 11]. IBD has been linked to an imbalance in microbial communities, termed gut dysbiosis, characterized by decreased microbial diversity owing to a shift in the balance between commensal and potentially pathogenic bacteria. Specifically, the phylum of Firmicutes, such as *Faecailbacterium prausnitzii*, is significantly reduced in the gut microbiota of patients with IBD [4]. Conversely, the phylum of Proteobacteria, particularly the family of Enterobacteriaceae, is commonly increased in IBD patients compared to non-IBD healthy individuals [4]. Although cause–effect mechanistic relationships have been challenging in human IBD, pre-clinical animal studies support a crucial role of the gut microbiota in the pathogenesis of IBD by utilizing gnotobiotic mouse models. For example, the colonization by the gut microbiota from IBD patients into germ-free (GF) mice increases the numbers of specific helper T cell subsets, including Th17 and Th2 cells, and decreases the numbers of the retinoid orphan receptor gamma t (RORγt)^+^ regulatory T (Treg) cells compared to the microbiota from healthy individuals [12]. Indeed, colonization by the gut microbiota from patients with IBD causes gut inflammation in models of colitis [12, 13]. In contrast to the dysbiotic microbiota, the healthy human gut microbiota does not cause gut inflammation even in genetically susceptible IBD-prone mice [13]. Additionally, transplanting healthy donor-derived microbiota into the mice colonized with human IBD microbiota induces RORγt^+^ Treg cells, which are protected against gut inflammation [14], suggesting that dysbiotic gut microbiota potentially contributes to gut inflammation by augmenting pro-inflammatory immune response. Consistent with animal studies, clinical trials of FMT have shown some efficacy in patients with IBD [6]. Therefore, modification of the gut microbiota is considered a valuable strategy for the treatment of IBD. In this regard, probiotics and prebiotics are useful microbiota-management tools for improving host health. Probiotics are specific viable microorganisms, such as *Lactobacillus* and *Bifidobacterium*, that may confer health benefits. In contrast, prebiotics are fermentable carbohydrates metabolized by gut microbes to beneficial metabolites, such as short-chain fatty acids and indole derivatives. Although the efficacy of probiotics and prebiotics are observed in several animal studies [15–17], there is little clinical evidence to support the effectiveness of this practice in patients with IBD [18]. To improve the efficacy of probiotics, recent studies have developed engineered probiotics that sense inflammatory regions and produce therapeutic molecules [19]. For example, oral administration of *L. lactis* secreting an anti-TNF nanobody attenuates gut inflammation in the murine model colitis [20]. Furthermore, oxygen-tolerant *F. prausnitzii,* by utilizing the cross-feeding system with *Desulfovibrio piger,* has recently developed [21]. Although there is little clinical evidence for these next-generation probiotics in IBD, these probiotics can potentially promote personalized medicine in IBD treatment.

#### Pathobionts in IBD

**Fig. 1:**
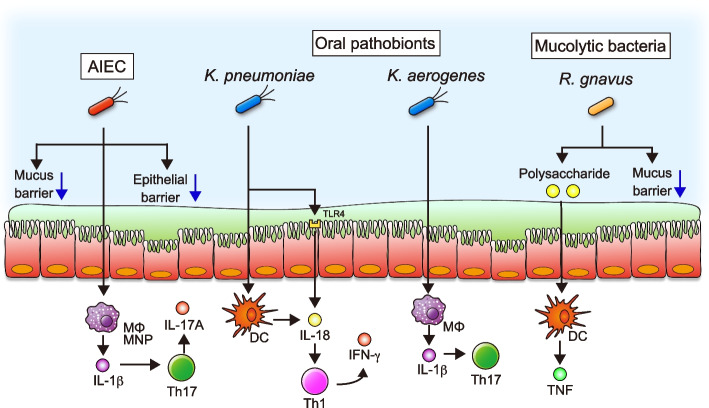
The mechanisms of gut inflammation caused by colonization of IBD-associated pathobionts. AIEC can adhere to and invade the epithelial cells, impairing the epithelial barrier. After invasion to the host, AIEC triggers IL-1β secretion from mononuclear phagocytes, such as macrophages, promoting the differentiation to Th17 cells. Oral pathobionts, including *K. pneumoniae* and *K. aerogenes*, can ectopically colonize the gut during gut inflammation. Ectopic colonization of oral pathobionts promotes the production of proinflammatory cytokines from DCs and macrophages, which facilitate the differentiation into Th1 and Th17 cells. Mucolytic bacteria, such as *R. gnavus*, may promote the encroachment of other bacteria to the epithelial niche. *R. gnavus* also produces the polysaccharide that promotes tumor necrosis factor (TNF) secretion from DCs

Certain members of the gut microbiota with pathogenic potentials, namely pathobionts, are considered to contribute closely to the pathogenesis of IBD (Fig. 1) [22, 23]. Pathobionts are commensal microorganisms that can cause gut inflammation under specific environmental or genetic influences. For example, adherent–invasive *Escherichia coli* (AIEC) has been proposed as a pathobiont based on its ability to degrade intestinal mucus and adhere and invade intestinal epithelial cells (IECs) [24]. A recent systematic review has reported that the prevalence of AIEC is higher in both CD and UC patients than in healthy individuals [25], supporting the claim that AIEC is involved in IBD. Unlike pathogenic *E. coli*, the colonization of AIEC induces none or only mild gut inflammation in healthy mice [26]. However, in genetically susceptible mice and chemically induced colitis mice, AIEC colonization results in massive gut inflammation and fibrosis [26, 27]. In this regard, pro-inflammatory cytokines up-regulate carcinoembryonic antigen-related cell adhesion molecule 6 (CEACAM6) receptors in IECs [28]. Adhesion to IECs is mediated by type 1 pili expressed on the surface of AIEC via interaction with CEACAM6 [28, 29]. Notably, CEACAM6 is overexpressed in the mucosa of CD than in healthy individuals, enhancing the colonization of AIEC in the intestinal mucosa of CD [28]. AIEC also degrades intestinal mucus by a serine protease called VAT-AIEC, which promotes the colonization of ileal and colonic mucosa [30]. After interacting with IECs, AIEC impairs the intestinal epithelial barrier by re-organizing the molecules involved in the epithelial barrier, such as zonula occludens-1 (ZO-1) and E-cadherin. Furthermore, AIEC colonization drives the production of IL-1b in mononuclear phagocytes, which augments the Th17 inflammatory response and develops gut inflammation [31]. Thus, the accumulation of AIEC in the intestinal mucosa contributes to the pathogenesis of IBD.

Recent studies have demonstrated a close association between oral-resident bacteria and the pathogenesis of IBD [32, 33]. Extraintestinal manifestations (EIMs) occur in up to 40% of IBD patients, and the oral cavity is a common site for EIMs in patients with IBD [34, 35]. Earlier studies highlighted a higher prevalence of periodontitis in patients with IBD than in controls without IBD [36]. Consistent with gut dysbiosis, the microbial communities in the oral cavity are also changed in patients with IBD [37]. Interestingly, oral-resident bacteria, such as Fusobacteriaceae, Pasteurellaceae, and Veillonellaceae, are enriched in the intestinal mucosa of treatment-naïve pediatric IBD patients [38, 39], indicating ectopically colonization of oral bacteria in the gut of patients with IBD. In the context of oral pathobionts, the strains of *Klebsiella* species, including *Klebsiella pneumoniae* and *K. aeromobilis,* isolated from the saliva of CD patients were reported to cause gut inflammation (Fig. 1) [40]. Mechanistically, the colonization by oral-derived *Klebsiella* species in GF mice induces Th1 cells via Toll-like receptor (TLR) and IL-18 signaling in dendritic cells (DCs) and IECs. Transcriptomic analysis shows that colonization of *K. pneumoniae* upregulates IFN-inducible (IFI) genes, such as guanylate-binding proteins (GBPs), chemokine (C-X-C motif) ligand 9 (Cxcl9), and dual oxidase 2 (Duox2), in DCs and IECs. These results imply that the IFI-mediated feed-forward loop regulates Th1 responses triggered by *K. pneumoniae* [40]. Likewise, the recent large cohort studies also found that *K. pneumoniae* is enriched in the feces of IBD patients, and colonization by isolated *K. pneumoniae* strain can also induce Th1 response [41]. Additionally, an animal study demonstrated that oral inflammation caused by ligature-induced periodontitis expands *Klebsiella* and *Enterobacter* species in the oral cavity, which in turn translocate to the inflamed gut [42]. Ectopic colonization of these oral pathobionts promotes IL-1β production via activation of inflammasome in macrophages, leading to the development of colitis in genetically susceptible mice.

In the context of other potential pathobionts for IBD, the accumulation of mucolytic bacteria, including *Ruminococcus gnavus* and *R. torque*, in mucosal tissues of IBD patients has been reported [43]. As intestinal mucus acts as a physical barrier against luminal antigens, including resident microbiota, disruption of the mucus barrier promotes the penetration of luminal antigens, resulting in gut inflammation [44, 45]. Thus, mucus degradation by mucolytic bacteria may facilitate the colonization of other bacteria in the epithelial niche. Additionally, *R. gnavus* produces an inflammatory polysaccharide that induces TNF secretion via TLR4 in DCs [46]. Although the mono-colonization of *R. gnavus* has fewer impacts on T cell response, *R. gnavus* has the potential to promote Th17 response by cooperating with *E. coli* [47]. In addition to mucolytic bacteria, a recent study found that *Clostridium innocuum* translocates into the mesenteric adipose tissue of CD patients and promotes the formation of creeping fat, which is associated with the development of intestinal fibrosis and structuring [48]. Interestingly, isolated *C. innocuum* strains are functionally and genetically distinct from luminal strains, as they are adapted to metabolize lipids and β-hydroxybutyrate. Translocated *C. innocuum* in mesenteric adipose tissue stimulates tissue remodeling via M2-like macrophages, expanding mesenteric adipose tissue [48].

These studies suggest that potentially pathogenic bacteria are intimately involved in the pathogenesis of IBD. Therefore, microbiome-based therapy targeting IBD-associated pathobionts is critical for the treatment of IBD. To date, dietary modification, bacteriophage, and IgA targeting pathobionts have been proposed to suppress pathobionts colonization [41, 49, 50]; however, no human studies have yet been conducted.

## Metabolic network between host and gut microbiota

### The host metabolism shapes gut microbiota

Recent studies suggest that host metabolism shapes gut microbiota by regulating the luminal microenvironment [51]. Consistent with host cells, the synthesis of adenosine triphosphate (ATP) is essential for bacterial growth. To generate ATP through the redox reactions, the electrons are transferred from the electron donors to the electron acceptors, such as oxygen and nitrate. The host regulates the availability of electron acceptors and controls bacterial communities in the gastrointestinal tract. For example, in the gastrointestinal tract, oxygen levels in the lumen gradually decrease from the duodenum to the colon and maintain a hypoxic condition in the colon [52]. In the large intestine, oxygen is consumed by the host IECs through mitochondria oxidative phosphorylation [51], and thus, the large intestine is maintained in the condition of physiological hypoxia. As a result, obligate anaerobic bacteria belonging to the classes of Bacteroidia and Clostridia dominate the microbial community in the large intestine [53]. In contrast to the large intestine, the ileum features the synthesis of superoxide and nitric oxide via epithelial NADPH oxidase (NOX1) and inducible nitric oxide synthase (iNOS), which decomposes to nitrate in the ileal lumen [54]. Accordingly, the microbial community in the ileum is dominated by facultatively anaerobic bacteria, including the class of Bacilli and the order of Enterobacterales. In the absence of host NOX1 or iNOS, microbial communities in the ileum are like microbial communities in the cecum [55]. Conversely, neither NOX1 nor iNOS influences microbial communities in the large intestine [55], suggesting that host-derived NOX1 and iNOS regulate microbial communities in the ileum but not the large intestine under a steady state. This evidence indicates that the host metabolism controls the luminal microenvironment, selecting which metabolic groups dominate the gut microbiota.

### The host metabolism during inflammation alters gut microbiota

**Fig. 2:**
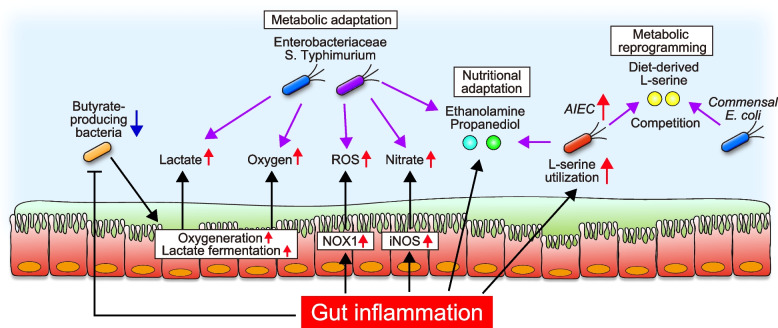
The role of host-microbe interaction in expanding pathobionts and pathogens during gut inflammation. Gut inflammation changes the host epithelial metabolism, which provides nutrients and electron acceptors for the expansion of pathobionts and pathogens. Pathobionts and pathogens also utilize unique mucosal nutrients, such as ethanolamine and propanediol, that commensals cannot use for growth. In the inflamed gut, AIEC up-regulates L-serine metabolism and operates diet-derived L-serine for competitive fitness with commensal *E. coli*

Healthy gut microbiota is characterized by the dominance of obligate anaerobic bacteria, including the phyla Firmicutes and Bacteroidetes. In contrast, the expansion of facultative anaerobic Enterobacteriaceae is commonly associated with gut dysbiosis [11]. Gut dysbiosis is triggered by antibiotic therapy, a Western-style diet, or certain diseases, including IBD [10]. Certain pathogenic bacteria and Enterobacteriaceae can bloom during gut inflammation by adapting to the inflammatory microenvironment (Fig. 2). For example, iNOS is highly expressed in the inflamed gut, and elevated concentrations of nitric oxide are observed in patients with UC [56, 57]. The reaction of nitric oxide radicals with superoxide radicals yields peroxynitrite, which can generate nitrate [51]. Unlike obligate anaerobic bacteria, facultative anaerobic bacteria, including Enterobacteriaceae, can utilize nitrate as terminal electron acceptors for anaerobic respiration [58]. The fitness of the *E. coli* strain that lacks the genes associated with nitrate utilization in the inflamed gut is lower than wild-type strain [58]. Importantly, nitrate utilization has a minimal effect on its fitness in the healthy gut, indicating that *E. coli* acquires a growth advantage by utilizing nitrate only during gut inflammation. In this context, nitrate utilization does not influence the colonization of *E. coli* in the inflamed gut of iNOS-deficient mice, suggesting that *E. coli* utilizes inflammation-driven host nitrate for the colonization in the inflamed gut [58]. In addition to Enterobacteriaceae, an IBD-associated oral pathobiont utilizes nitrate for ectopic colonization in the gut during intestinal inflammation. A recent study found that *Veillonella* species, obligate anaerobes present in the human oral cavity, are enriched in the gut of IBD patients [59]. They usually obtain energy by fermenting short-chain organic acids, such as lactate and malate. However, *V. parvula* changes its metabolism from fermentation to nitrate respiration for ectopic colonization in the inflamed gut. In addition, nitrate respiration modulates the metabolic repertoire of *V. parvula*, allowing it to use amino acids and peptides as energy sources. This metabolic reprogramming promotes ATP synthesis through oxidative phosphorylation, boosting the growth of *V. parvula* under nitrate respiration [59].

Similarly, increased luminal oxygen levels induced by gut inflammation or gut dysbiosis also contribute to the expansion of Enterobacteriaceae and pathogens [60]. For example, the depletion of butyrate-producing bacteria by broad-spectrum antibiotic treatment increases epithelial oxygenation in the large intestine, expanding the facultative anaerobic bacteria, including Enterobacteriaceae and pathogens [61]. In the context of mechanism, butyrate produced by clostridia activates peroxisome proliferator-activated receptor-γ (PPAR-γ) signaling in IECs, which in turn limits the availability of luminal oxygen by driving the energy metabolism of IECs toward b-oxidation [61]. Indeed, butyrate treatment enhances oxygen consumption in intestinal epithelial cell lines, which stabilizes hypoxia-inducible factor (HIF), a transcription factor regulating the intestinal barrier [62]. Also, the supplementation of tributyrin, an analog of butyrate, can restore antibiotics-induced epithelial oxygenation, which protects the intestinal barrier via HIF [62]. Depletion of short-chain fatty acid (SCFA)-producing clostridia also promotes lactate fermentation through PPARγ-signaling in IECs [63]. Increased availability of luminal lactate induced by antibiotics treatment boosts the colonization of *Salmonella enterica* serovar Typhimurium in a lactate utilization-dependent manner.

These studies indicate that the metabolic connection between the host and gut microbiota regulates the expansion of certain pathogenic bacteria during gut inflammation. The inflammatory microenvironment gives pathobionts and pathogenic bacteria growth advantage in the gut, leading to positive feedback between inflammation and dysbiosis. Resolving inflammation can suppress the expansion of pathogenic bacteria in vivo; however, pathobionts still exist in some IBD patients even in remission, suggesting that pathobionts may have other strategies to colonize in the gut of IBD patients.

## Metabolic and nutritional adaptation of pathobionts to inflammatory microenvironment

### Metabolic reprogramming

Pathobionts and pathogens have evolved various strategies to overcome competition with commensals during gut inflammation. One of the essential strategies is bacterial metabolic reprogramming, adapted to the inflammatory microenvironment. For example, host immune activation reprograms the transcription of metabolic genes and metabolic activities in commensal bacteria within several hours [64]. Additionally, gut inflammation upregulates stress-response pathways and downregulates polysaccharide utilization and fermentation in microbial communities of colitis mice [65]. In addition, commensal *E. coli* up-regulates stress-response genes, including small heat-shock proteins, in response to chronic intestinal inflammation [66].

In the context of IBD-associated pathobionts, AIEC reprograms its metabolic function to adapt to the inflammatory microenvironment (Fig. 2). In the healthy gut, AIEC mainly utilizes sugar as an energy source. Conversely, gut inflammation up-regulates L-serine metabolism pathways, which are crucial in acquiring a growth advantage over commensal *E. coli* strains [49]. L-serine is a non-essential amino acid and a central hub of metabolism in disease conditions. For example, cancer cells and activated immune cells up-regulate L-serine utilization, and they use L-serine for their proliferation [67–70]. Interestingly, these cells utilize L-serine supplied from the diet, and therefore, the restriction of dietary L-serine can suppress the proliferation of these cells [68, 69]. Consistently, the deprivation of dietary L-serine can regulate intraspecific competition between AIEC and commensal *E. coli* strains in the inflamed gut [49]. Therefore, IBD-associated pathobionts reprogram their metabolism for expansion in the inflamed gut. This metabolic reprogramming can be viewed as therapeutic targets that selectively inhibit the growth of certain pathogenic bacteria without influencing beneficial commensal bacteria.

### Nutritional adaptation

Pathobionts and pathogens utilize unique nutrients, such as ethanolamine and 1,2-propanediol, that commensal symbionts cannot use to overcome competition with commensals. For example, S. Typhimurium has been known to utilize ethanolamine, which is abundant in the intestine, for its growth. In the presence of tetrathionate as a respiratory electron acceptor, ethanolamine supports the anaerobic growth of S. Typhimurium [71]. Indeed, the fitness of the S. Typhimurium strain that lacks the gene involved in ethanolamine utilization in the inflamed gut is lower than wild-type strain [71], suggesting that ethanolamine utilization confers a growth advantage of S. Typhimurium during gut inflammation. Likewise, unlike non-AIEC strains, IBD-associated AIEC strains can utilize ethanolamine as a source of nitrogen and carbon [72, 73]. Specific metabolites, such as bile acids and propionate, regulate the ethanolamine utilization genes of AIEC [72, 74]. In addition, ethanolamine utilization regulates virulence genes associated with bacterial motility, adhesion and invasion to IECs, and proinflammatory response, which augments the pathogenicity of AIEC [73]. AIEC also utilizes fucose and propanediol as a carbon source through the *pdu* operon, which is part of a metabolic pathway involved in fucose metabolism [75]. In *E. coli* species, pduC (propanediol dehydratase) regulates the conversion of 1,2-propanediol to propionaldehyde, which is ultimately converted to propionate [75]. Notably, pduC-encoding AIEC is expanded in CD patients and induces colitis through IL-1β produced by CX_3_C motif chemokine receptor 1 (CX3CR1)^+^ mononuclear phagocyte [31]. As the mechanism, pduC is required to produce downstream metabolite propionate, and pduC-dependent propionate production by AIEC drives IL-1β secretion through NLRP3- and Caspase 11-dependent inflammasome activation in macrophages. Interestingly, inhibition of mucosal fucosylation by a (1,2)-fucosylation inhibitor 2-deoxy-D-galactose limits AIEC-induced colitis [31], suggesting that the intestinal mucosa is a unique source of nutrients including the fucose and 1,2-propanediol. Notably, these metabolites are enriched in the ileal mucosa of CD patients [73], suggesting that AIEC utilizes the metabolites for the colonization of the gut of CD patients. Selectively using specific nutrients gives pathobionts a competitive advantage over commensal bacteria.

## Nutritional crosstalk between commensals and pathobionts/pathogens

### Direct metabolic network

**Fig. 3:**
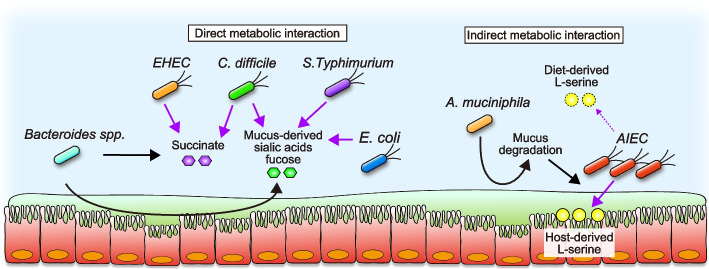
Direct and indirect metabolic interaction between commensals, pathobionts, and pathogens. Commensal Bacteroides spp. provide succinate and host-mucus derived sialic acids and fucose for the expansion of pathogenic bacteria, including EHEC, *C. difficile*, S. Typhimurium, and commensal *E. coli* (Direct metabolic network). Mucolytic bacteria, such as *A. muciniphila*, degrade intestinal mucus, promoting the encroachment of AIEC to the intestinal epithelium. In the epithelial niche, AIEC liberates host-derived L-serine for expansion under L-serine deficient conditions (Indirect metabolic network)

In addition to host-microbe interaction, nutritional interactions between gut microbes shape microbial communities (Fig. 3). These interactions include competition, syntropy, cross-feeding, and commensal bacteria control the pathogens through these strategies. One strategy for indigenous microbial communities to eliminate pathogenic bacteria is the preferential consumption of nutrients required to grow competing pathogenic bacteria. For example, commensal *E. coli* competes with pathogenic *E. coli*, including enterohaemorrhagic *E. coli* (EHEC) and enteropathogenic *E. coli* (EPEC), for carbohydrates, organic acids, amino acids, and other nutrients [76–79]. Likewise, the colonization of *Phascolarctobacterium* reduces the availability of luminal succinate, a crucial metabolite for the growth of *Clostridioides difficile*, preventing the growth of *C. difficile* [80]. By consuming nutritional resources, commensal microbes can cause the starvation of competing pathogens, preventing the expansion of pathogens. On the other hand, some commensal bacteria provide nutrient resources that pathogens utilize for expansion in the gut. *Bacteroides thetaiotaomicron*, a member of the Bacteroidetes phylum and major constituent of the microbiota, encodes several glycoside hydrolases and polysaccharide lyases, and *B. thetaiotaomicron*-degraded complex polysaccharides can readily be used by other bacteria. For example, S. Typhimurium and *C. difficile* share a common strategy of degrading mucosal glycans liberated by *B. thetatiotaomicron* during their expansion in the gut [81]. Precisely, *B. thetatiotaomicron* liberates sialic acids from host mucin and increases the availability of luminal sialic acids, which is utilized by S. Typhimurium and *C. difficile* for the bloom in the gut. Likewise, as a nutrient source, *C. difficile* utilizes succinate generated by *B. thetatiotaomicron*-colonized mice [82]. These host-derived glycans serve not only as a nutrient source but also as signaling molecules that regulate the virulence genes of pathogens. For instance, EHEC senses the fucose liberated by *B. thetaiotaomicron* from the host mucus, modulating the expression of the virulence factor Ler, a master regulator of the locus of enterocyte effacement (LEE) genes in EHEC [83]. These studies suggest that a beneficial member of the microbiota has the capacity to promote the growth and virulence of pathogens.

### Indirect metabolic network

Commensal bacteria not only provide nutritional resources but also indirectly enhance the growth and virulence of pathogens and pathobionts by promoting association with host cells under certain conditions. For example, diet-derived fibers maintain the intestinal mucus barrier by preventing the expansion of mucolytic bacteria. In the gnotobiotic mice colonized by the consortium of commensal bacteria that mimic human gut microbiota, the deprivation of dietary fibers promotes the expansion of mucolytic bacteria, including *B. thetaiotaomicron*, *B. caccae*, *Barnesiella intestinihominis*, and *Akkermansia muciniphila* *[*84*]*. These mucolytic bacteria impair the intestinal mucus barrier by consuming the mucus layer, increasing susceptibility to the mucosal pathogen *Citrobacter rodentium*. In another context, mucolytic bacteria also promote the growth of IBD-associated AIEC under dietary L-serine restriction [85]. As mentioned above, AIEC utilizes diet-derived L-serine for the expansion in the inflamed gut by up-regulating L-serine metabolism [49]; however, dietary L-serine restriction leads to the expansion of AIEC and subsequent exacerbation of colitis in specific pathogen-free (SPF) mice. Interestingly, dietary L-serine restriction promotes the abnormal expansion of AIEC only when it coexists with mucolytic bacteria, such as *A. muciniphila*. Notably, *A. muciniphila* facilitates the encroachment of AIEC to the epithelial niche by degrading the mucus barrier. In the epithelial niche, AIEC acquires L-serine pooled in the host colonic epithelium to counteract dietary L-serine restriction, suggesting that mucolytic bacteria, such as *A. muciniphila*, can serve as an indirect metabolic supporter for AIEC by licensing the acquisition of host-derived nutrients [85]. Given that metabolic support by commensal bacteria influences the pathogenicity of pathobionts, the therapeutic intervention targeting pathobionts also needs to consider the impact of co-existing commensal bacteria.

## Metabolic network of the gut microbiota as therapeutic targets in IBD

### The role of microbial metabolites in intestinal homeostasis

Given the contribution of microbial metabolism in the pathogenesis of IBD, it is plausible that such metabolites are valuable targets for treating IBD. In this regard, accumulating evidence suggests that microbial fiber and tryptophan metabolites play a key role in intestinal homeostasis [86, 87]. For example, SCFAs, such as acetate, propionate, and butyrate, are the major byproducts of dietary fibers by microbial fermentation. In addition to the role of energy substrates, SCFAs act as signaling molecules via G-protein-coupled receptors (GPRs) and regulate the differentiation of immune cells [3]. In this context, SCFAs, particularly butyrate, are well-known molecules that regulate gene expression epigenetically by inhibiting histone deacetylases (HDACs). For example, the inhibition of HDACs by butyrate induces histone H3 acetylation in naive T cells, which in turn, up-regulates the Foxp3 expression and promotes the differentiation of Treg [88]. Indeed, GF mice lacking the fiber-fermented microbiota display lower levels of colonic Foxp3+ Treg cells [89]. Consistent with the animal studies, fiber-fermented bacteria, including *F. prausnitzii* and *Roseburia hominis*, and fecal levels of SCFAs are lower in IBD than those in healthy controls [90]. Furthermore, recent studies highlight that tryptophan metabolites, including indole-3-aldehyde and indole-3-acetic acid, play crucial roles as regulators of immunity [91]. Tryptophan undergoes metabolism into indole derivatives by specific gut bacteria, including *Lactobacillus* and *Clostridium*, signaling to the aryl hydrocarbon receptor (AhR) [86]. Mice lacking CARD9, a susceptibility gene for IBD, exhibit reduced numbers of *Lactobacillus* strains capable of metabolizing tryptophan. This deficiency leads to impaired AhR-mediated IL-22 production, rendering them susceptible to colitis [92]. In line with animal experiments, AhR activity and tryptophan metabolites are reduced in IBD patients, especially in those carrying the CARD9 risk alleles associated with IBD [92]. Collectively, gut dysbiosis linked to IBD alters luminal metabolites, subsequently impacting mucosal immunity.

### Future directions for microbiota-targeted therapy in IBD

Given the crucial role of microbial metabolites in mucosal immunity, there is therapeutic potential for treating IBD through directly supplementing metabolites themselves or indirectly via metabolite source nutrients and probiotic bacteria. Numerous animal studies have demonstrated that supplementation with metabolites, including SCFAs and AhR ligands, have anti-inflammatory properties in mouse models of colitis [88, 92, 93]. However, in contrast to animal studies, there is little evidence in the clinical setting. In this regard, the function and composition of the gut microbiota in IBD patients is heterogeneous, requiring personalized approaches adapted to the characteristics of genetic factors, clinical background, and gut microbiota. For example, dietary fibers are typically beneficial for gut health; however, some patients with IBD report intolerance to fiber consumption. A recent study shows that unfermented β-fructan fibers induce pro-inflammatory responses in intestinal immune cells from a subset of IBD patients [94]. The adverse impact of dietary fiber in specific IBD patients is likely linked to a deficiency in fermentative microbial activities. This implies that the microbial fermentative potential plays a crucial role in influencing the efficacy of fiber supplementation. Likewise, reduced production of AhR ligands is observed in the microbiota of certain IBD patients, particularly in those with CARD9 risk alleles [92]. Thus, supplementation with tryptophan or AhR ligands may be more effective in patients who have lower activity of AhR.

Additionally, the metabolic network between commensals-pathobionts may influence treatment efficacy. In particular, the pathogenic capacity of pathobionts, such as AIEC, is context-dependent. For example, the restriction of dietary L-serine can suppress the expansion of AIEC [85]. However, when mucolytic bacteria co-exist with AIEC, dietary L-serine deprivation promotes the bloom of AIEC in the gut by facilitating the encroachment of AIEC to the epithelial niche, resulting in the exacerbation of colitis [85]. This suggests that pathobionts can induce detrimental effects only in the presence of metabolic supporters. Thus, complex metabolic interactions determine the success of gut microbiota-targeted interventions.

## Conclusion

Accumulating evidence supports the notion that IBD-associated pathobionts are associated with the pathogenesis of IBD. Over the past decade, several pathobionts have been identified from the oral and gut microbiota of patients with IBD. However, in contrast to the development of biological agents, the evidence regarding the therapeutic efficacy of targeting gut microbiota, such as FMT and probiotics, has not been sufficient in IBD. In this regard, the metabolic network between host-microbe or commensals-pathobionts may influence treatment efficacy. In the context of IBD, clinical and translational studies show that the metabolic activity of gut microbiota influences the response to the biological agent and dietary intervention [94, 95]. To effectively treat IBD, a personalized treatment tailored to an individual’s gut microbiota is required. This next generation of treatment-targeted gut microbiota will require the selection of patients who will respond and non-respond to the treatment. Therefore, a better understanding of host-microbe interactions and the development of predictive biomarkers will help to identify subgroups of IBD patients.

## Data Availability

Not applicable.

## Abbreviations

IBD: Inflammatory bowel disease
UC: Ulcerative colitis
CD: Crohn’s disease
GF: Germ-free
RORγt: Retinoid orphan receptor gamma t
Treg: Regulatory T cells
FMT: Fecal microbiota transplantation
AIEC: Adherent invasive Escherichia coli
IECs: Intestinal epithelial cells
CEACAM6: Carcinoembryonic antigen-related cell adhesion molecule 6
DCs: Dendritic cells
TLR: Toll-like receptor
IFI: Interferon γ-inducible
TNF: Tumor necrosis factor
ATP: Adenosine triphosphate
NOX1: NADPH oxidase 1
iNOS: Inducible nitric oxide synthase
HIF: Hypoxia-inducible factor
SCFAs: Short-chain fatty acids
